# The relationship between psychological safety, patient safety culture, and work engagement with patient safety outcomes: a cross-sectional study in a Japanese university hospital

**DOI:** 10.1186/s12913-026-14257-z

**Published:** 2026-03-02

**Authors:** Takeo Hata, Masahiko Nitta, Takami Matsuo, Kohei Arai, Eiichiro Ueda, Masami Nishihara, Akira Ashida, Masashi Neo, Takahiro Katsumata, Masaaki Hoshiga

**Affiliations:** 1 Department of Pharmacy, Osaka Medical and Pharmaceutical University Hospital, 2-7 Daigaku-machi, Takatsuki, Osaka 569-8686 Japan; 2 Department of Hospital Quality and Safety Management, Osaka Medical and Pharmaceutical University Hospital, 2-7, Daigaku-machi, Takatsuki, Osaka 569-8686 Japan; 3 Graduate School of Business Administration, Kobe University, 2-1 Rokkodai-cho, Nada- ku, Kobe, 657-8501 Japan; 4 Graduate School of Business, Osaka Metropolitan University, 1-1 Gakuen-cho, Naka- ku, Sakai, 599-8531 Japan; 5 Department of Pediatrics, Faculty of Medicine, Osaka Medical and Pharmaceutical University, 2-7, Daigaku-machi, Takatsuki, Osaka 569-8686 Japan; 6 Department of Orthopedic Surgery, Faculty of Medicine, Osaka Medical and Pharmaceutical University, 2-7, Daigaku-machi, Takatsuki, Osaka 569-8686 Japan; 7 Department of Thoracic and Cardiovascular Surgery, Faculty of Medicine, Osaka Medical and Pharmaceutical University, 2-7 Daigaku-machi, Takatsuki, Osaka 569- 8686 Japan; 8 Department of Cardiology, Faculty of Medicine, Osaka Medical and Pharmaceutical University, 2-7 Daigaku-machi, Takatsuki, Osaka 569-8686 Japan

**Keywords:** Healthcare, Incident, Patient safety, Patient safety culture, Psychological safety, Work engagement

## Abstract

**Background:**

Patient safety is a central concern in healthcare, as adverse events result in prolonged hospital stays, increased costs, and serious harm to patients. Previous research has highlighted the importance of patient safety culture, psychological safety, and work engagement, but evidence linking these organizational characteristics with objective safety indicators is limited. This study aimed to clarify the relationships among patient safety culture, psychological safety, work engagement, and incident counts as an objective measure of patient safety.

**Methods:**

This single-center, cross-sectional study included all employees of a Japanese university hospital as of April 2021. A questionnaire survey was conducted to assess patient safety culture, psychological safety, and work engagement. Patient safety culture was measured using the Hospital Survey on Patient Safety Culture, psychological safety using Edmondson’s Team Psychological Safety scale, and work engagement using the Utrecht Work Engagement Scale. Incident counts reported between 2018 and 2020 were used as the patient safety indicator. Factor structures were validated with factor analyses. Relationships among factors were examined using multilevel structural equation modeling at both the individual and department levels, with multiple regression analyses performed as sensitivity analyses.

**Results:**

Of 3,316 distributed questionnaires, 2,734 responses were received (82.4% response rate), and 1,500 were included in the final multilevel structural equation modeling analysis. Factor analyses supported the 12-factor model of patient safety culture, a one-factor model of psychological safety, and a one-factor model of work engagement. Psychological safety was positively associated with patient safety culture and work engagement at both individual and department levels. At the department level, psychological safety showed the strongest negative association with incident counts (standardized estimate of − 0.67). Work engagement was not significantly related to incident counts. Sensitivity analyses excluding outlier departments yielded consistent results.

**Conclusions:**

Psychological safety was strongly associated with improved patient safety culture and higher work engagement, and it was the factor most strongly linked to lower incident counts. These findings suggest that enhancing psychological safety among healthcare staff is an effective strategy for reducing adverse events and improving patient safety. Future research should focus on interventions to foster psychological safety across diverse healthcare settings.

**Supplementary Information:**

The online version contains supplementary material available at 10.1186/s12913-026-14257-z.

## Background

Patient safety is the most critical element in healthcare. It is estimated that medical errors in hospitals result in the deaths of approximately 98,000 Americans annually [1]. Additionally, population-based data from the Colorado and Utah Medical Practices Study suggest that preventable adverse events in the outpatient setting in the United States lead to 75,000 hospitalizations each year, with 4,839 cases resulting in permanent disability and 2,587 deaths [2]. A systematic review of reports on inpatient adverse events revealed that 9.2% of patients experienced adverse events, of which 43.5% were preventable, and 7.4% were fatal [3]. Unintended adverse events are a significant issue for individual patients, hospitals, and society because they lead to readmissions [2], prolonged hospital stays [4], increased healthcare costs [4, 5], and severe disabilities or deaths [4, 6]. More recent studies have confirmed that patient safety remains a global healthcare challenge, with preventable adverse events continuing to impose substantial clinical and economic burdens across healthcare systems worldwide [7, 8].

Cultivating a positive patient safety culture is crucial for enhancing the quality and safety of healthcare [9]. Facilities with a positive patient safety culture have been reported to experience fewer medical errors [10], shorter hospital stays [11], and lower mortality rates [11]. Additionally, such facilities exhibit lower readmission rates within 30 days for acute myocardial infarction or heart failure [12], fewer surgical site infections [13], and reduced rates of postoperative complications and pressure ulcers [14]. Moreover, a positive patient safety culture is associated with lower levels of staff burnout [15].

Psychological safety, a concept proposed by Edmondson [16], is defined as the shared belief that a team is safe for interpersonal risk-taking. There is evidence that hospital staff, despite feeling specific concerns about patient safety, observing potential errors, or noticing rule violations, often remain silent [17, 18]. Notably, higher levels of perceived support for patient safety and psychological safety are significantly associated with reduced frequencies of withholding voice [17]. Furthermore, a cross-sectional study of nurses working in Dutch hospitals found a positive correlation between nurses’ willingness to speak up and team psychological safety [19]. Thus, psychological safety is essential for promoting open communication in healthcare settings.

Studies on the effects of psychological safety in healthcare often report positive outcomes. Nurses’ psychological safety positively correlates with their responsibility for preventing infection transmission [20]. For emergency department physicians, internal incivility is associated with low psychological safety [21]. Incivility, defined as a facet of broader negative communication, involves ignoring colleagues and fostering situations of contempt, conflict, and stress. O’Donovan et al. emphasized the need for initiatives that promote effective communication among healthcare professionals to support patient safety culture, and for nurse managers to support nurses’ psychological safety [22]. These findings suggest that psychological safety is closely related to patient safety.

Work engagement is defined as a positive, fulfilling, work-related state of mind characterized by vigor, dedication, and absorption [23]. A systematic review of 417 studies investigating predictors of turnover intentions among Korean nurses found that work engagement was negatively associated with turnover intentions [24]. Regarding patient safety, some studies report a negative relationship between nurses’ work engagement and the risk of patient falls [25], while others found no significant association [26]. These findings suggest that higher work engagement may reduce turnover among hospital staff and improve work quality. However, the effect of work engagement on patient safety remains unclear.

Identifying organizations best suited for safer healthcare is valuable. However, although previous studies suggest that patient safety culture, psychological safety, and work engagement are related to patient safety, unified results are lacking. Additionally, a limitation of these studies is the use of surrogate safety indicators. Patient safety is influenced by factors operating at multiple levels within healthcare organizations. Patient safety culture reflects organizational norms and management practices related to safety, psychological safety captures the interpersonal climate that enables speaking up and learning within teams, and work engagement represents individual-level motivation and involvement in work. Together, these constructs span organizational, team, and individual domains that have been theoretically and empirically linked to safety-related behaviors and outcomes. This multilevel perspective is consistent with established frameworks in safety science and organizational psychology, including safety culture frameworks and socio-technical systems approaches, as well as job demands–resources models that conceptualize work engagement as a key individual-level factor influencing performance and safety-related behaviors. Focusing on these interrelated constructs allows for a multilevel examination of how organizational context and workforce characteristics jointly contribute to patient safety. Importantly, while prior studies have examined pairwise relationships between psychological safety and work engagement or patient safety culture, most relied on self-reported or surrogate safety outcomes. In contrast, this study simultaneously examines patient safety culture, psychological safety, and work engagement across organizational, team, and individual levels, using department-level incident report counts as an objective patient safety indicator. Therefore, this study aims to elucidate the characteristics that enhance patient safety by examining the relationships between patient safety culture, psychological safety, work engagement, and the objective patient safety indicator of incident counts.

## Methods

### Study design

We conducted a single-center, cross-sectional observational study. The design combined an anonymous questionnaire survey of hospital employees with secondary analysis of incident report data. This study targeted all employees of a Japanese university hospital as of April 1, 2021. The hospital, located in Takatsuki City, Osaka Prefecture, serves as a core general hospital with 31 clinical departments and 832 beds. As a university hospital, it provides advanced medical care, which entails significant patient safety risks and potentially involves various types of risks. The hospital has continuously engaged in activities to enhance the quality and safety of healthcare and has established a culture of actively submitting incident reports. Thus, it is a logical research site, offering high-quality data collection.

### Data sources

To measure patient safety culture, psychological safety, and work engagement, we conducted a survey among the study subjects and used their responses for analysis. Incident counts were used as the patient safety indicator. Incident counts included in the analysis were those reported in incident reports from 2018 to 2020, excluding near misses, and included only incidents that had some impact on patients.

### Questionnaire

The survey included questions to measure patient safety culture, psychological safety, and work engagement. For patient safety culture, we used the AHRQ Hospital Survey on Patient Safety Culture (HSOPSC) [27] from the United States Agency for Healthcare Research and Quality (AHRQ). This survey consists of 42 items that form a model of 12 subscales related to patient safety. The HSOPSC has been utilized and validated in various countries outside the United States [28, 29]. For psychological safety, we used Edmondson’s Team Psychological Safety scale [16], which comprises 7 items. For work engagement, we used the Japanese version of the Utrecht Work Engagement Scale (UWES-J) [30], created by Shimazu et al., based on Schaufeli’s Utrecht Work Engagement Scale (UWES) [23, 31]. This scale consists of 9 items that form a model of 3 subscales. For HSOPSC and Psychological Safety, the forward translation into Japanese was performed independently by two authors with expertise in patient safety and organizational psychology. Discrepancies between the two provisional translations were reconciled through discussion. A professional bilingual translator (Cactus Communications Pvt. Ltd.) then conducted a back-translation into English, and the research team compared the back-translated version with the original items to ensure semantic equivalence. Before the full survey, we conducted a small pilot test with hospital staff (*n* = 5) to evaluate clarity, comprehension, and cultural appropriateness of the translated items. The final English versions of all HSOPSC and Psychological Safety items are provided in Tables S1 and S2.

### Exclusion criteria

In this study, we established the following exclusion criteria: multiple selections for single-choice questions, any unanswered items, inappropriate responses, and logical inconsistencies in responses regarding the background of the respondents. We included a question requiring a selection of 5 on a 5-point Likert scale; respondents who selected any other option were considered to have provided inappropriate responses and were excluded. Respondents were also excluded for logical inconsistencies if they selected a non-physician profession but chose a clinical department (which only physicians can belong to) as their department.

### Data processing

Responses to negatively worded items, also known as reverse-coded items, were reverse-coded before analysis. Specifically, for responses on a 5-point Likert scale, 1 was converted to 5, 2 to 4, 4 to 2, and 5 to 1. For responses on a 7-point Likert scale, 1 was converted to 7, 2 to 6, 3 to 5, 5 to 3, 6 to 2, and 7 to 1.

No organizational restructuring (merging or splitting of departments) occurred between 2018 and 2021. A few wards underwent relocation and name changes during this period; however, these represented administrative renaming only, and the underlying departmental structure, staffing, and reporting lines remained unchanged. For analyses, such units were treated as the same department, and incident reports before and after relocation were combined accordingly.

### Statistical analysis

We conducted confirmatory factor analysis to verify whether the response data for the scales of patient safety culture, psychological safety, and work engagement obtained in this study fit existing models. If the fit to existing models was found to be low, exploratory factor analysis was performed to extract factors to be used as variables in subsequent analyses. Estimates were obtained using the maximum likelihood estimation method. To compare each factor score based on respondents’ backgrounds, we drew heatmaps using column z-scores, calculated by subtracting the column mean from each respondent’s value and dividing by the column standard deviation. To confirm the necessity of multilevel modeling, we calculated the intraclass correlation coefficient (ICC) for the incident rate. The ICC was 0.156, indicating substantial between-department variability and supporting the use of multilevel models. Using multilevel structural equation modeling, we examined the relationships among patient safety culture, psychological safety, work engagement, and the number of incidents at both the individual and department levels. To account for differences in department size, the incident counts were standardized by dividing the total number of incidents in each department (2018–2020) by the number of staff members belonging to that department at the time of the 2021 survey. This exposure adjustment was performed to avoid confounding between the absolute number of incidents and departmental size. The standardized incident rate (incidents per staff member) was used as the outcome variable in all department-level analyses. For sensitivity analysis, we conducted three additional analyses. (1) To examine the potential influence of outliers on the multilevel structural equation modeling results, we re-ran the model after excluding the four departments with extremely high numbers of incident reports. (2) To address the temporal mismatch between the survey (April 2021) and the incident data, we re-estimated the multilevel structural equation model using only incident reports from 2020 instead of the aggregated data from 2018 to 2020. (3) We conducted multiple regression analysis to evaluate the relationship between the factors and the incident rate. All reported *p*-values were 2-tailed, and the level of significance was set at *p* < 0.05. Statistical analyses were performed using R version 4.0.2 (R Development Core Team, Vienna, Austria).

### Descriptive statistics of responses

The descriptive statistics for responses to questions related to the scales of patient safety culture, psychological safety, and work engagement are shown in Tables S3–S5. Among the 9 items of the work engagement scale, a ceiling effect was observed for item I3.

### Validation of the 12-factor model of patient safety culture using confirmatory factor analysis

We examined whether the data from this study fit the 12-factor model [27] of the patient safety culture scale using confirmatory factor analysis (Table S6). The comparative fit index (CFI) was 0.911, the Tucker–Lewis index (TLI) was 0.899, the root mean square error of approximation (RMSEA) was 0.046, and the standardized root mean square residual (SRMR) was 0.041, indicating no issues with fit. CFI ranges from 0 to 1, with values closer to 1 indicating better fit; generally, CFI of 0.95 or higher is considered indicative of a good model. TLI also indicates better fit as it approaches 1 and can sometimes exceed 1. RMSEA of 0.05 or below suggests a good fit, while values of 0.1 or above indicate poor fit. SRMR has a lower bound of 0, with values closer to 0 indicating better fit. The factor loadings for the 42 items ranged from 0.452 to 0.956, supporting the model’s fit to the data. Internal consistency indices were calculated for each of the 12 subscales of the patient safety culture scale. Cronbach’s alpha values ranged from 0.63 to 0.87, and McDonald’s omega values ranged from 0.64 to 0.88 (Table S7). Although several subscales showed moderate internal consistency (α = 0.63–0.69), the overall reliability was considered acceptable for research use, particularly given that the original 12-factor structure demonstrated good fit in the confirmatory factor analysis. Therefore, we determined that the 12-factor model of the patient safety culture scale was appropriate for our data and used these 12 factors (f1–f12) as variables.

### Validation of the one-factor model of psychological safety using confirmatory factor analysis

We examined whether the data from this study fit the one-factor model [16] of psychological safety using confirmatory factor analysis (Table S8). CFI was 0.926, TLI was 0.889, RMSEA was 0.112, and SRMR was 0.054, indicating a poor fit. Item H1 (“If you make a mistake on this team, it is often held against you.”) showed a negative factor loading, indicating that it did not align with the latent construct measured by the other six items. In Edmondson’s original scale, this item captures “interpersonal risk-taking related to asking for help”, which may behave differently in hierarchical healthcare contexts. Therefore, we attempted to extract factors using exploratory factor analysis.

### Exploration of the psychological safety model using exploratory factor analysis

Based on the Kaiser–Guttman criterion, eigenvalues, the scree plot (Figure S1), and the results of the parallel analysis (Figure S2), we determined that a one-factor solution was appropriate. After excluding item H1 and re-running the analysis, the factor loadings ranged from 0.601 to 0.842, as shown in Table S9, and a single factor was extracted. Reliability indices were high (Cronbach’s alpha = 0.84; McDonald’s omega = 0.85). Removing H1 improved factor structure and internal consistency. Because the remaining six items cover the core dimensions of team psychological safety (e.g., openness, risk-taking, mutual respect), the impact on content validity is considered limited. Therefore, for psychological safety, we used the one-factor model consisting of the six items (H2–H7) as a latent variable (f13).

### Validation of the three-factor model of work engagement using confirmatory factor analysis

We first examined whether the data from this study fit the conventional three-factor model of work engagement [23, 30, 31] using confirmatory factor analysis (Table S10). Although the factor loadings were generally high, the overall model fit indices indicated a poor fit: CFI was 0.909, TLI was 0.863, RMSEA was 0.180, and SRMR was 0.062. This pattern is consistent with prior Japanese validation research [30], which demonstrated that, in Japan, the original three subscales of vigor, dedication, and absorption tend to collapse into a single engagement factor. Shimazu et al. further reported that the nine-item UWES-J exhibited superior fit to the 17-item version and that a one-factor model achieved the best fit across multiple samples. These findings suggest that the multidimensional UWES structure may not be optimal in Japanese organizational contexts, likely due to high intercorrelations among the three components of engagement. Given both the poor fit of the three-factor model in our data and prior evidence supporting a unidimensional structure in Japan, we conducted exploratory factor analysis.

### Exploration of the work engagement model using exploratory factor analysis

Exploratory factor analysis indicated that a one-factor solution was appropriate based on the Kaiser–Guttman criterion, scree plot (Figure S3), and parallel analysis (Figure S4). Item I3 (“I am enthusiastic about my job.”) exhibited a pronounced ceiling effect, a phenomenon also observed in Japanese samples [30]. After excluding I3, the one-factor solution showed strong and consistent factor loadings ranging from 0.715 to 0.925 (Table S11). Reliability indices were excellent (Cronbach’s alpha = 0.95; McDonald’s omega = 0.95). Importantly, the remaining eight items still cover all three conceptual domains—vigor, dedication, and absorption—indicating that the exclusion of I3 did not compromise content validity. These findings align with a previous study of the UWES-J [30], which concluded that the one-factor model provides the most appropriate representation of the UWES-J in Japanese occupational settings. Therefore, work engagement was operationalized as a single latent factor composed of eight items (I1, I2, and I4–I9) for use in our multilevel structural equation modeling (f14).

## Results

### Analysis subjects

**Fig. 1:**
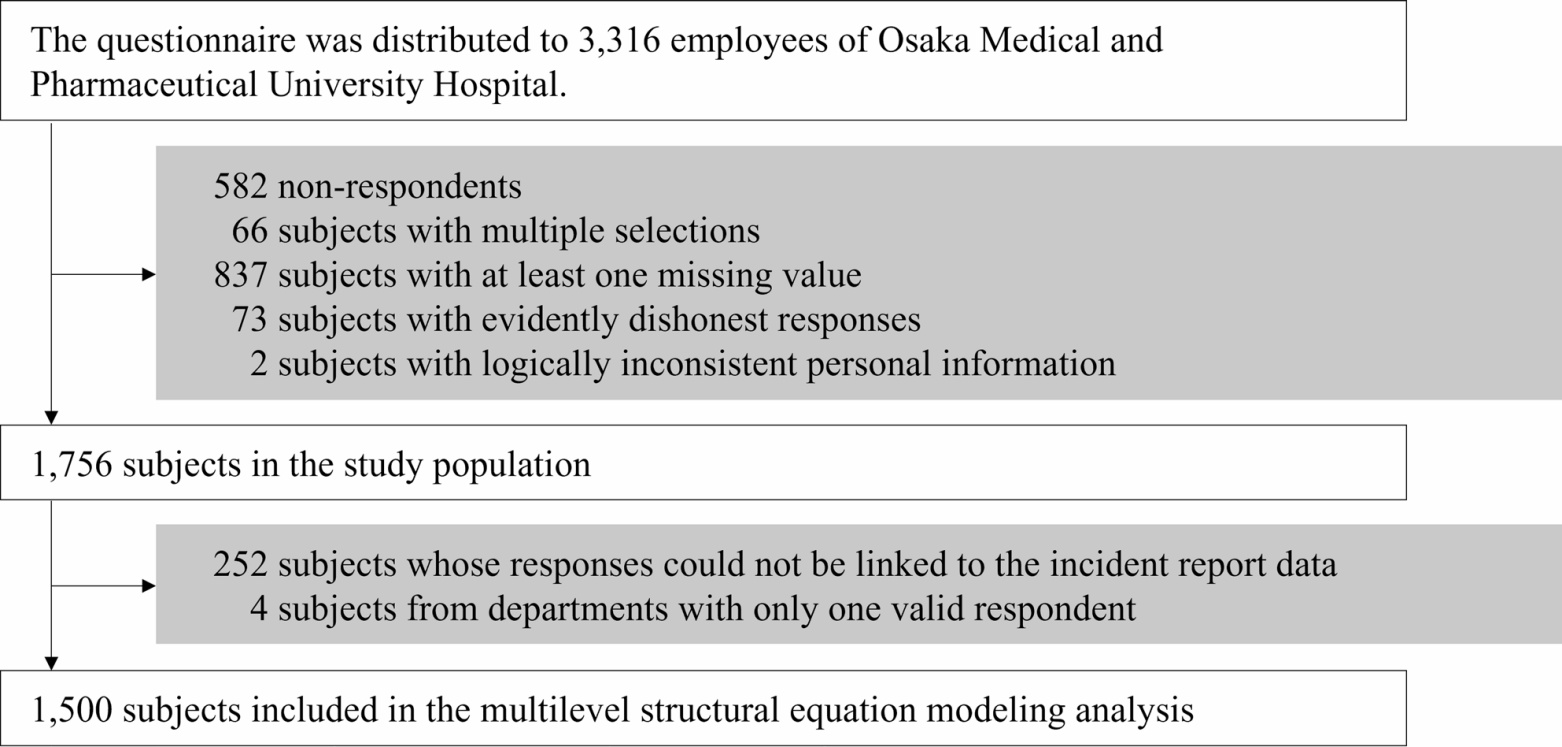
Flow diagram for the selection of the study population

The selection flow of analysis subjects is shown in Fig. 1. Questionnaires were distributed to 3,316 employees, and responses were received from 2,734 (response rate: 82.4%). After applying the exclusion criteria, 1,756 respondents were selected as subjects for analyses excluding multilevel structural equation modeling. Of these, 252 respondents whose responses could not be linked to the incident report data using their department as a key, and 4 respondents who were the sole members of their departments, were excluded. Thus, 1,500 respondents were included in the analysis using multilevel structural equation modeling. The incident reports used as the patient safety indicator in the analysis totaled 6,685.

### Backgrounds of study population

The background of the analysis subjects is shown in Table 1. Nurses comprised the largest group at 43.1%, followed by Physicians/Dentists at 19.0%, Medical Staff at 15.0%, and Unit Assistants/Clerks/Secretaries also at 15.0%. The Medical Staff category included pharmacists, physical therapists, and clinical laboratory technicians. Females made up 74.0% of the respondents, approximately three times the number of males (26.0%). The age group of 20 to under 30 years accounted for 41.6% of the respondents. Regular staff members comprised 75.0%. Regarding years of practical experience in their current specialty or profession, 23.1% had 3 to 5 years. For years of service at this hospital, 27.6% had 1 to 2 years, and for years of service in their current hospital work area/unit, 36.3% had 1 to 2 years. The average weekly working hours were 40–59 h for 49.5% of the respondents.

**Table 1:** Backgrounds of study population

Characteristics	Frequency	Proportion (%)
What is your staff position in this hospital?		
Physician/Dentist	334	19.0
Nurse	756	43.1
Medical Staff	263	15.0
Unit Assistant/Clerk/Secretary	263	15.0
Other	140	8.0
Gender		
Male	457	26.0
Female	1,299	74.0
Age (year)		
< 20	11	0.6
20–29	731	41.6
30–39	413	23.5
40–49	316	18.0
≥ 50	285	16.2
Working form		
Regular Staff Member	1,317	75.0
Temporary Employee	27	1.5
Consignment Staff	96	5.5
Dispatched Workers	54	3.1
Part-time Staff	97	5.5
Other	165	9.4
How long have you worked in your current specialty or profession?		
1–2 years	403	22.9
3–5 years	406	23.1
6–10 years	355	20.2
11–20 years	360	20.5
≥ 21 years	232	13.2
How long have you worked in this hospital?		
1–2 years	484	27.6
3–5 years	461	26.3
6–10 years	332	18.9
11–20 years	316	18.0
≥ 21 years	163	9.3
How long have you worked in your current hospital work area/unit?		
1–2 years	638	36.3
3–5 years	487	27.7
6–10 years	322	18.3
11–20 years	216	12.3
≥ 21 years	93	5.3
Typically, how many hours per week do you work in this hospital?		
< 20 h	146	8.3
20–39 h	577	32.9
40–59 h	870	49.5
60–79 h	109	6.2
≥ 80 h	54	3.1
Are you a manager currently?		
Yes	151	8.6
No	1,605	91.4
In your staff position, do you typically have direct interaction or contact with patients?		
Yes	1,586	90.3
No	170	9.7
In your staff position, do you typically have direct care for patients?		
Yes	1,152	65.6
No	604	34.4

Percentages within each characteristic category may not sum to exactly 100% because of rounding.; *n* = 1,756

### Descriptive statistics of each factor score

The factor scores for each of the 14 factors were calculated as the average of the responses to the items associated with each factor. These scores were standardized for each factor to produce column z-scores, which were then compared based on respondents’ backgrounds (Figure S5). For Physicians/Dentists, Psychological Safety (f13) and Communication Openness (f7) scores were higher, while for Nurses, Feedback & Communication About Error (f6), Frequency of Events Reported (f8), and Handoffs & Transitions (f11) scores were higher. In contrast, Medical Staff showed lower scores for Psychological Safety (f13) and Teamwork Within Units (f1). Overall, factor scores tended to decrease with age, although Work Engagement (f14) increased in the 40–49 and 50 years and older age groups. Across different lengths of experience in their job, hospital, or department, the scores were highest in the 1–2 years range and tended to decrease with more years of experience. However, Work Engagement (f14) dropped sharply after three years and then increased with additional years of experience. Longer average weekly working hours were associated with lower factor scores. Except for Staffing (f10), managerial positions had higher factor scores. Those who had opportunities to interact with patients or provide direct care during work had generally higher factor scores, although Staffing (f10) was an exception, showing lower scores. Notably, Psychological Safety (f13) tended to be lower for those without patient interaction opportunities. The correlation matrix among all factor scores is provided in Table S12.

### Validation of research hypotheses using multilevel structural equation modeling

**Fig. 2:**
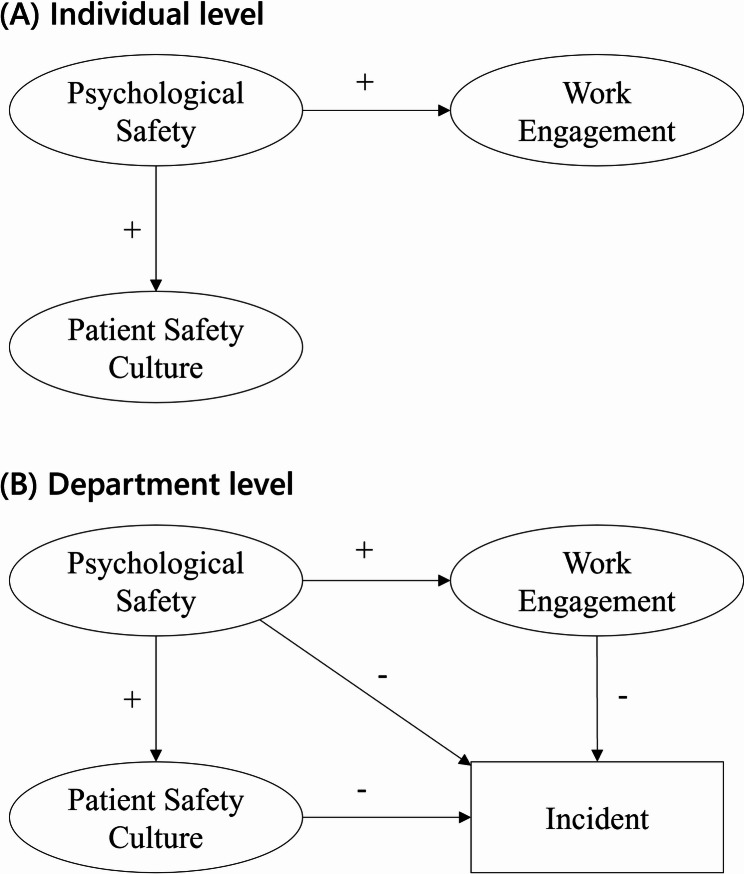
Research hypothesis model. In the figure, a plus sign indicates a positive correlation, while a minus sign indicates a negative correlation

We examined the relationships between psychological safety, patient safety culture, work engagement, and the number of incidents using multilevel structural equation modeling. Analyses were conducted at both the individual level and the department level. We hypothesized that psychological safety is related to patient safety culture and work engagement, and that at the department level, these factors are further related to the number of incidents. This was the basis for our research hypothesis model (Fig. 2). At the individual level, variables were centered within the cluster, meaning the values represented the deviation of individual scores from the group mean. At the department level, group mean values were used as variables. The analysis results indicated no issues with model fit, with comparative fit index (CFI) of 0.964, Tucker–Lewis index (TLI) of 0.910, root mean square error of approximation (RMSEA) of 0.057, and standardized root mean square residual (SRMR) of < 0.001 (individual level) and 0.162 (department level). However, the SRMR value was high at the department level.

The individual-level results are shown in Table 2. Psychological Safety (f13) had significant associations with the 12 factors of patient safety culture (f1–f12). The standardized estimates were notably high for Communication Openness (f7) at 0.63 and Teamwork Within Units (f1) at 0.61. Additionally, Psychological Safety (f13) was significantly related to Work Engagement (f14), with a standardized estimate of 0.43.

**Table 2:** Analysis of inter-factor relationships by multilevel structural equation modeling (individual level)

Affecting variables		Affected variables	Estimates	SE	95%CI	Standardized estimates	*p*-value
13. Psychological Safety	→	1. Teamwork Within Units	0.41	0.01	0.38–0.44	0.61	< 0.001*
		2. Supervisor/Manager Expectations & Actions Promoting Patient Safety	0.36	0.01	0.33–0.39	0.55	< 0.001*
		3. Organizational Learning―Continuous Improvement	0.28	0.01	0.25–0.30	0.50	< 0.001*
		4. Management Support for Patient Safety	0.33	0.02	0.30–0.36	0.47	< 0.001*
		5. Overall Perceptions of Patient Safety	0.33	0.02	0.30–0.36	0.50	< 0.001*
		6. Feedback & Communication About Error	0.39	0.02	0.35–0.42	0.52	< 0.001*
		7. Communication Openness	0.51	0.02	0.48–0.54	0.63	< 0.001*
		8. Frequency of Events Reported	0.26	0.02	0.22–0.31	0.29	< 0.001*
		9. Teamwork Across Units	0.36	0.02	0.32–0.39	0.48	< 0.001*
		10. Staffing	0.23	0.02	0.20–0.27	0.32	< 0.001*
		11. Handoffs & Transitions	0.28	0.02	0.25–0.31	0.43	< 0.001*
		12. Nonpunitive Response to Errors	0.41	0.02	0.37–0.45	0.48	< 0.001*
13. Psychological Safety	→	14. Work Engagement	0.79	0.04	0.70–0.87	0.43	< 0.001*

SE, standard error; CI, confidence interval; Comparative fit index (CFI), 0.964; Tucker-Lewis index (TLI), 0.910; Root mean square error of approximation (RMSEA), 0.057; Standardized root mean square residual (SRMR), 0.000 (within); *, *p* < 0.05; *n* = 1,500

The department-level results are shown in Table 3. At the department level, Psychological Safety (f13) also had significant associations with the 12 factors of patient safety culture (f1–f12). The standardized estimates were notably high for Communication Openness (f7) at 0.82, Supervisor/Manager Expectations & Actions Promoting Patient Safety (f2) at 0.79, Nonpunitive Response to Errors (f12) at 0.76, Teamwork Within Units (f1) at 0.69, and Organizational Learning―Continuous Improvement (f3) at 0.69. Additionally, Psychological Safety (f13) was significantly related to Work Engagement (f14), with a standardized estimate of 0.58. In examining the relationship with the number of incidents, among the 12 factors of patient safety culture (f1–f12), Staffing (f10) showed a negative association with the number of incidents, with a standardized estimate of -0.20. Conversely, positive associations with the number of incidents were observed for Supervisor/Manager Expectations & Actions Promoting Patient Safety (f2) at 0.59, Handoffs & Transitions (f11) at 0.46, Teamwork Within Units (f1) at 0.33, and Feedback & Communication About Error (f6) at 0.32. Psychological Safety (f13) had the most substantial negative association with the number of incidents, with a standardized estimate of -0.67. Work Engagement (f14) was not associated with number of incidents.

**Table 3:** Analysis of inter-factor and incident count relationships by multilevel structural equation modeling (department level)

Affecting variables		Affected variables	Estimates	SE	95%CI	Standardized estimates	*p*-value
13. Psychological Safety	→	1. Teamwork Within Units	0.44	0.05	0.35–0.53	0.69	< 0.001*
		2. Supervisor/Manager Expectations & Actions Promoting Patient Safety	0.53	0.04	0.45–0.61	0.79	< 0.001*
		3. Organizational Learning―Continuous Improvement	0.40	0.04	0.31–0.48	0.69	< 0.001*
		4. Management Support for Patient Safety	0.28	0.05	0.18–0.38	0.48	< 0.001*
		5. Overall Perceptions of Patient Safety	0.45	0.05	0.35–0.54	0.68	< 0.001*
		6. Feedback & Communication About Error	0.43	0.07	0.30–0.55	0.55	< 0.001*
		7. Communication Openness	0.56	0.04	0.48–0.64	0.82	< 0.001*
		8. Frequency of Events Reported	0.33	0.08	0.17–0.49	0.38	< 0.001*
		9. Teamwork Across Units	0.23	0.06	0.11–0.35	0.35	< 0.001*
		10. Staffing	0.19	0.09	0.02–0.37	0.21	0.033*
		11. Handoffs & Transitions	0.34	0.06	0.22–0.46	0.50	< 0.001*
		12. Nonpunitive Response to Errors	0.55	0.05	0.46–0.65	0.76	< 0.001*
13. Psychological Safety	→	14. Work Engagement	0.98	0.14	0.70–1.25	0.58	< 0.001*
1. Teamwork Within Units	→	Number of incidents	3.09	1.16	0.82–5.36	0.33	0.008*
2. Supervisor/Manager Expectations & Actions Promoting Patient Safety			5.25	1.18	2.94–7.57	0.59	< 0.001*
3. Organizational Learning―Continuous Improvement			-0.13	1.43	-2.92–2.67	-0.01	0.929
4. Management Support for Patient Safety			-1.26	1.62	-4.45–1.92	-0.12	0.436
5. Overall Perceptions of Patient Safety			0.00	1.38	-2.71–2.71	0.00	1.000
6. Feedback & Communication About Error			2.46	1.03	0.44–4.47	0.32	0.017*
7. Communication Openness			-1.72	1.39	-4.44–1.00	-0.20	0.215
8. Frequency of Events Reported			-0.31	0.68	-1.64–1.01	-0.05	0.646
9. Teamwork Across Units			-0.67	1.13	-2.89–1.55	-0.07	0.554
10. Staffing			-1.30	0.61	-2.48–-0.11	-0.20	0.032*
11. Handoffs & Transitions			4.08	1.07	1.99–6.17	0.46	< 0.001*
12. Nonpunitive Response to Errors			-1.28	1.17	-3.57–1.02	-0.16	0.275
13. Psychological Safety			-3.99	1.17	-6.27–-1.71	-0.67	0.001*
14. Work Engagement			-0.04	0.35	-0.74–0.65	-0.01	0.901

SE, standard error; CI, confidence interval; Comparative fit index (CFI), 0.964; Tucker-Lewis index (TLI), 0.910; Root mean square error of approximation (RMSEA), 0.057; Standardized root mean square residual (SRMR), 0.162 (between); *, *p* < 0.05; *n* = 98

### Sensitivity analysis

#### Exclusion of outlier departments.

To account for the potential influence of outliers on the results of the multilevel structural equation modeling (Tables 2 and 3), we re-ran the analysis excluding the four departments with an extremely high number of incidents (Figure S6) and compared the results to those obtained in the main analysis. The results remained consistent with the main analysis, showing that Psychological Safety (f13) had statistically significant associations with the 12 factors of patient safety culture (f1–f12) and Work Engagement (f14), and it had the most substantial negative association with the number of incidents (Tables S13 and S14).

#### Use of 2020 incident reports only

As a second sensitivity analysis, we re-ran the multilevel structural equation model using only incident reports from 2020 as the outcome. Model fit indices remained acceptable (CFI = 0.964, TLI = 0.908, RMSEA = 0.058, SRMR_(individual level) < 0.001, SRMR_(department level) = 0.158). At the individual level, Psychological Safety (f13) was again significantly associated with all 12 factors of patient safety culture (f1–f12), with standardized estimates ranging from 0.29 to 0.64, and with Work Engagement (f14; standardized estimate 0.42) (Table S15). At the department level, Psychological Safety (f13) remained positively associated with all 12 factors of patient safety culture (standardized estimates 0.23–0.85) and with Work Engagement (f14; standardized estimate 0.61). Consistent with the main analysis, Staffing (f10) showed a negative association with the incident rate, whereas Teamwork Within Units (f1), Supervisor/Manager Expectations & Actions Promoting Patient Safety (f2), and Handoffs & Transitions (f11) showed positive associations. Psychological Safety (f13) again showed the strongest negative association with the incident rate (standardized estimate of − 0.94) (Table S16). These results indicate that the main findings are robust when incidents are restricted to a single year proximal to the survey.

#### Multiple regression analysis

Finally, we examined the relationships among the 12 factors of patient safety culture (f1–f12), Psychological Safety (f13), Work Engagement (f14), and the number of incidents at the department level using multiple regression analysis. The results were consistent with those obtained in the main analysis (Table S17).

## Discussion

In this study, we investigated which organizational characteristics enhance patient safety, focusing on patient safety culture, psychological safety, and work engagement. Previous research often used surrogate measures as patient safety indicators, such as the number of patient falls or the perceptions of physicians or nurses regarding patient safety and the quality of work. Reports using actual incident count data as a patient safety indicator were scarce. This study is likely one of the few that uses incident counts as a patient safety indicator. Our analysis revealed that psychological safety positively correlated with both patient safety culture and work engagement. Furthermore, psychological safety was identified as the factor most strongly associated with a lower number of incidents.

Although prior studies have suggested that higher psychological safety may lead to increased reporting of near misses and adverse events [32], our findings showed that psychological safety had the strongest association with a reduction in the incident rate and was positively correlated with both patient safety culture and work engagement. This suggests that psychological safety may not only contribute to improving patient safety but also enhance employees’ positive and fulfilling engagement with their work. This interpretation is consistent with recent evidence indicating that psychological safety is associated with reductions in missed nursing care and lower turnover intention among hospital nurses [33]. The association observed in our study also aligns with earlier work showing that psychological safety can reduce patient safety risks through improved communication among clinical teams; for example, Dietl et al. demonstrated in obstetric care settings that psychological safety promotes communication, which in turn mitigates patient safety risks [34]. Furthermore, our findings are in line with a growing body of research highlighting the role of psychological safety in supporting effective teamwork and patient safety practices. A recent study of emergency department nurses reported a linear association between psychological safety, teamwork, and patient safety competency [35], reinforcing the importance of cultivating psychologically safe work environments to enhance the quality of care. Conversely, the previous study focusing on healthcare workers in intensive care units found that higher psychological safety was independently associated with higher perceptions of teamwork but was not related to the incident rates of spontaneous breathing trials and lung-protective ventilation [36]. This indicates that while psychological safety is related to modifiable factors, or latent variables, it does not directly correlate with the actual practice of evidence-based procedures [36]. While our study targeted incidents across the entire hospital, future research should investigate which specific types of incidents are primarily influenced by psychological safety. This could provide more detailed insights into the impact of psychological safety on different aspects of patient safety.

In this study, no association was found between work engagement and the patient safety indicator. This suggests that while improving employees’ motivation and reducing turnover rates can enhance the relationship between work and the individual, patient safety must be addressed as a separate issue. A survey comparing hospitals that experienced major adverse events related to infection control with those that did not found that the former had lower employee engagement and psychological safety [37]. Employee engagement refers to a state where employees have a good relationship with their organization, are satisfied with their work, and are enthusiastic about their job [38]. Work engagement is defined as a positive, fulfilling, work-related state of mind and does not include the relationship between employees and the organization [23]. Therefore, to enhance patient safety, it may be more important to improve the relationship between employees and the organization rather than just focusing on the relationship between employees and their work. High work engagement appears to be associated with stronger patient safety culture, although evidence regarding safety outcomes remains limited [15].

In the results of this study, there were mixed associations between factors of patient safety culture and the patient safety indicator. Previous research has shown that higher average scores for Organizational Learning―Continuous Improvement (f3) are significantly associated with lower rates of pressure ulcers, long-term physical restraints, and complaints. Similarly, higher average scores for Frequency of Events Reported (f8) are significantly associated with lower rates of medical errors and pressure ulcers [39]. On the other hand, a survey of nurses working in Swiss acute care hospitals found no significant association between patient safety culture and seven patient outcomes (nurse-reported medication errors, pressure ulcers, patient falls, urinary tract infections, bloodstream infections, pneumonia, and patient satisfaction) [40]. In this study, several patient safety culture subscales—such as Teamwork Within Units (f1), Supervisor/Manager Expectations & Actions Promoting Patient Safety (f2), and Feedback & Communication About Error (f6)—showed positive associations with the incident rate. These subscales contain items that directly assess communication around errors, expectations for reporting, and collaborative climate, all of which are closely linked to reporting-related behaviors. Higher scores on these dimensions may therefore reflect a more active reporting culture rather than a true increase in adverse events. This interpretation is consistent with the HSOPSC technical guidance from AHRQ [27], which notes that stronger reporting and feedback processes often lead to higher observed reporting volumes. At the same time, it is important to acknowledge that patient safety culture is a multidimensional construct, and the relationship between individual subscales and incident reporting may vary depending on the type of event and the functional characteristics of each department. Continued research is needed to disentangle the aspects of safety culture that influence actual safety performance from those that primarily promote reporting behavior.

Zhou et al. analyzed differences in perceptions of patient safety culture across different work units and professions, suggesting that strategies and measures to improve the patient safety environment need to be adjusted based on the department and profession of the employees [41]. In this study, Psychological Safety (f13) was significantly lower among employees who were not physicians or nurses, had worked in their current department for 3 to 20 years, worked more than 40 h per week, were non-managers, or had no opportunities to interact with patients during work. Therefore, strategies to enhance psychological safety for these employees are necessary. Work Engagement (f14) was found to be low for employees with 3 to 10 years of experience in their current department, showing a recovery trend thereafter, likely due to improvements in position, benefits, or authority. In contrast, patient safety culture generally declined gradually after joining the organization, suggesting a negative correlation between work engagement and patient safety culture. To achieve a balance between work engagement and patient safety culture, it is important to identify the factors leading to a decline in patient safety culture after three years of employment. Changing departments through rotations could help maintain patient safety culture. Given that psychological safety and patient safety culture tend to decrease after three years in the same department, rotating departments every 2 to 3 years could be a reasonable strategy from a patient safety perspective.

This study analyzed data from all employees and incidents, making the results representative of the hospital. Additionally, using the objective measure of incident counts, rather than self-reported or perceived measures of patient safety, is a strength of this study. However, there are several limitations to consider. First, although confirmatory and exploratory factor analyses were conducted and internal consistency was assessed for all major constructs, the intrinsic validity of self-reported organizational and psychological measures remains an inherent limitation of this study. Patient safety culture, psychological safety, and work engagement are latent constructs that cannot be directly observed, and their measurement inevitably depends on respondents’ perceptions and interpretations. Accordingly, the associations identified in this study should be interpreted as relationships at the construct level rather than as definitive indicators of objective organizational performance or causal effects. Second, as a cross-sectional study, this research can only verify correlations between variables, not causal relationships. Third, the study uses incident report counts as a proxy for actual incident counts. While incident reporting systems are widely accepted and useful for collecting information on adverse events, they are prone to underreporting, which is a significant drawback [42]. Differences in underreporting rates between departments could introduce reporting bias. Additionally, multiple employees involved in a single incident may file separate reports, leading to potential duplicate reporting. Fourth, this is a single-center study, focusing on a university hospital. Therefore, the external validity or generalizability of the results to other settings must be considered carefully. Fifth, there was a temporal mismatch between the timing of the staff survey (April 2021) and the observation window for incident reports (2018–2020), which may have attenuated some associations. However, a sensitivity analysis restricted to incidents occurring in 2020 yielded results that were consistent with the main analysis. Sixth, the between-level SRMR was relatively high (0.162), suggesting suboptimal fit at the department level. This is likely due to unmeasured contextual factors such as case-mix, patient severity, clinical workload, or staffing patterns, which were not available in our institutional datasets. These variables are typically not collected in a standardized, department-level format in many hospital settings, including ours, and therefore could not be incorporated into the model. As a result, unexplained between-department variability may have inflated the between-level residuals. This limitation is common in organizational studies conducted in healthcare environments where detailed structural indicators at the unit level are often unavailable. Seventh, we acknowledge that some content overlap may exist between patient safety culture and psychological safety, particularly for items related to communication about errors and speaking up. In this study, however, patient safety culture and psychological safety were treated as conceptually distinct constructs and were assessed using separate instruments reflecting different levels of analysis: organizational norms and management practices (patient safety culture) versus team-level perceptions of interpersonal risk taking (psychological safety). We did not conduct a formal discriminant validity test using a joint measurement model including both item sets; therefore, the associations observed between these constructs should be interpreted with caution in light of potential conceptual proximity. Finally, there is the potential influence of common method variance (CMV), because the questionnaire-based variables (patient safety culture, psychological safety, and work engagement) were collected at a single time point and using the same response format. However, CMV is unlikely to fully account for the observed associations, because (1) the three constructs have distinct theoretical bases and demonstrated adequate discriminant validity in factor analyses, and (2) the outcome variable (incident rate) was derived from an independent administrative database rather than self-reported data. Nonetheless, CMV remains a possible source of bias and should be addressed in future longitudinal or multi-method studies.

## Conclusions

Psychological safety was positively correlated with both patient safety culture and work engagement. Incident counts were negatively correlated with psychological safety. This suggests that enhancing psychological safety is effective for improving patient safety. Future research in the medical field should focus on strategies to enhance psychological safety.

## Supplementary Information

Below is the link to the electronic supplementary material.

Supplementary Material 1

## Data Availability

The datasets generated and analyzed during the current study are not publicly available due to restrictions related to the privacy and confidentiality of hospital employees and patients. However, de-identified data may be available from the corresponding author on reasonable request.

## Abbreviations

AHRQ: Agency for Healthcare Research and Quality
CFI: comparative fit index
HSOPSC: Hospital Survey on Patient Safety Culture
RMSEA: root mean square error of approximation
SRMR: standardized root mean square residual
TLI: Tucker-Lewis index
UWES: Utrecht Work Engagement Scale
