# Supplementary material for: The relationship between psychological safety, patient safety culture, and work engagement with patient safety outcomes: a cross-sectional study in a Japanese university hospital
Source: BMC Health Serv Res. 2026 Mar 2;26:476. doi: 10.1186/s12913-026-14257-z (PMC13059359; doi:10.1186/s12913-026-14257-z)
Supplement: Supplementary file 1 — Supplementary Material 1 [file 12913_2026_14257_MOESM1_ESM.pdf]

Additional file

Supplemental Tables

Table S1 Final English version of the Patient Safety Culture Scale items

Codes	Items
Think about your hospital work area/unit…	
A1	People support one another in this unit.
A2	We have enough staff to handle the workload.
A3	When a lot of work needs to be done quickly, we work together as a team to get the work done.
A4	In this unit, people treat each other with respect.
A5	Staff in this unit work longer hours than is best for patient care.
A6	We are actively doing things to improve patient safety.
A7	We use more agency/temporary staff than is best for patient care.
A8	Staff feel like their mistakes are held against them.
A9	Mistakes have led to positive changes here.
A10	It is just by chance that more serious mistakes don't happen around here.
A11	When one area in this unit gets really busy, others help out.
A12	When an event is reported, it feels like the person is being written up, not the problem.
A13	After we make changes to improve patient safety, we evaluate their effectiveness.
A14	We work in "crisis mode" trying to do too much, too quickly.
A15	Patient safety is never sacrificed to get more work done.
A16	Staff worry that mistakes they make are kept in their personnel file.
A17	We have patient safety problems in this unit.
A18	Our procedures and systems are good at preventing errors from happening.
B1	My supervisor/manager says a good word when he/she sees a job done according to established patient safety procedures.
B2	My supervisor/manager seriously considers staff suggestions for improving patient safety.
B3	Whenever pressure builds up, my supervisor/manager wants us to work faster, even if it means taking shortcuts.
B4	My supervisor/manager overlooks patient safety problems that happen over and over.
Think about your hospital work area/unit…	
C1	We are given feedback about changes put into place based on event reports.
C2	Staff will freely speak up if they see something that may negatively affect patient care.
C3	We are informed about errors that happen in this unit.
C4	Staff feel free to question the decisions or actions of those with more authority.
C5	In this unit, we discuss ways to prevent errors from happening again.
C6	Staff are afraid to ask questions when something does not seem right.
D1	When a mistake is made, but is caught and corrected before affecting the patient, how often is this reported?
D2	When a mistake is made, but has no potential to harm the patient, how often is this reported?
D3	When a mistake is made that could harm the patient, but does not, how often is this reported?
Think about your hospital…	
F1	Hospital management provides a work climate that promotes patient safety.
F2	Hospital units do not coordinate well with each other.
F3	Things "fall between the cracks" when transferring patients from one unit to another.
F4	There is good cooperation among hospital units that need to work together.
F5	Important patient care information is often lost during shift changes.
F6	It is often unpleasant to work with staff from other hospital units.
F7	Problems often occur in the exchange of information across hospital units.
F8	The actions of hospital management show that patient safety is a top priority.
F9	Hospital management seems interested in patient safety only after an adverse event happens.
F10	Hospital units work well together to provide the best care for patients.
F11	Shift changes are problematic for patients in this hospital.

Table S2 Final English version of the Psychological Safety Scale items

Codes	Items
About the team you usually work with…	
H1	If you make a mistake on this team, it is often held against you.
H2	Members of this team are able to bring up problems and tough issues.
H3	People on this team sometimes reject others for being different.
H4	It is safe to take a risk on this team.
H5	It is difficult to ask other members of this team for help.
H6	No one on this team would deliberately act in a way that undermines my efforts.
H7	Working with members of this team, my unique skills and talents are valued and utilized.

Table S3 Descriptive statistics of responses to the patient safety culture survey

Codes	Items	Mean	SD	Proportion of responses (%)				
				1	2	3	4	5
Think about your hospital work area/unit…								
A1	People support one another in this unit.	4.07	0.71	0.6	2.9	9.3	63.2	24.0
A2	We have enough staff to handle the workload.	2.87	1.06	10.0	28.2	31.8	24.4	5.6
A3	When a lot of work needs to be done quickly, we work together as a team to get the work done.	3.97	0.71	0.5	3.9	12.5	64.7	18.5
A4	In this unit, people treat each other with respect.	3.88	0.79	1.1	4.8	16.6	59.9	17.7
A5*	Staff in this unit work longer hours than is best for patient care.	2.60	0.99	12.8	35.4	34.1	14.6	3.1
A6	We are actively doing things to improve patient safety.	4.03	0.62	0.2	1.5	12.1	67.9	18.3
A7*	We use more agency/temporary staff than is best for patient care.	3.44	1.00	3.4	13.4	32.7	36.5	13.9
A8*	Staff feel like their mistakes are held against them.	3.31	1.02	4.5	17.8	29.4	38.5	9.8
A9	Mistakes have led to positive changes here.	4.03	0.59	0.3	1.7	8.9	72.7	16.3
A10*	It is just by chance that more serious mistakes don't happen around here.	3.50	0.88	2.5	10.3	30.7	47.8	8.7
A11	When one area in this unit gets really busy, others help out.	3.65	0.92	4.1	7.3	19.2	58.0	11.3
A12*	When an event is reported, it feels like the person is being written up, not the problem.	3.34	0.92	2.9	14.9	34.8	39.9	7.5
A13	After we make changes to improve patient safety, we evaluate their effectiveness.	3.52	0.73	1.0	6.2	38.0	49.8	5.1
A14*	We work in "crisis mode" trying to do too much, too quickly.	3.25	0.91	2.9	17.4	37.1	36.8	5.8
A15	Patient safety is never sacrificed to get more work done.	3.54	0.87	1.4	11.4	29.2	48.1	10.0
A16*	Staff worry that mistakes they make are kept in their personnel file.	3.50	0.85	1.4	10.9	32.5	46.6	8.5
A17*	We have patient safety problems in this unit.	3.74	0.72	0.3	4.6	27.1	57.5	10.6
A18	Our procedures and systems are good at preventing errors from happening.	3.56	0.72	0.6	6.3	35.3	51.9	6.0
B1	My supervisor/manager says a good word when he/she sees a job done according to established patient safety procedures.	3.49	0.87	2.1	11.4	29.4	49.1	8.0
B2	My supervisor/manager seriously considers staff suggestions for improving patient safety.	3.83	0.75	1.0	4.4	18.7	62.0	13.8
B3*	Whenever pressure builds up, my supervisor/manager wants us to work faster, even if it means taking shortcuts.	4.06	0.77	0.5	3.5	13.7	54.6	27.7
B4*	My supervisor/manager overlooks patient safety problems that happen over and over.	4.00	0.75	0.6	3.0	15.5	58.0	22.8
Think about your hospital work area/unit…								
C1	We are given feedback about changes put into place based on event reports.	3.68	0.82	1.1	5.3	32.5	46.9	14.2
C2	Staff will freely speak up if they see something that may negatively affect patient care.	3.64	0.83	1.3	6.4	32.6	47.0	12.8
C3	We are informed about errors that happen in this unit.	3.91	0.80	0.9	3.6	20.9	52.8	21.9
C4	Staff feel free to question the decisions or actions of those with more authority.	3.56	0.90	1.7	9.6	32.9	42.3	13.5
C5	In this unit, we discuss ways to prevent errors from happening again.	3.83	0.80	0.9	3.6	25.8	51.0	18.7
C6*	Staff are afraid to ask questions when something does not seem right.	3.70	0.85	1.7	5.3	29.8	47.7	15.5
D1	When a mistake is made, but is caught and corrected before affecting the patient, how often is this reported?	3.81	0.89	0.3	8.0	24.9	44.0	22.8
D2	When a mistake is made, but has no potential to harm the patient, how often is this reported?	3.83	0.90	0.6	7.1	25.3	42.4	24.6
D3	When a mistake is made that could harm the patient, but does not, how often is this reported?	3.99	0.94	1.0	6.6	18.8	39.2	34.4
Think about your hospital…								
F1	Hospital management provides a work climate that promotes patient safety.	3.73	0.70	0.6	5.0	22.3	64.6	7.5
F2*	Hospital units do not coordinate well with each other.	3.18	0.89	2.7	20.0	37.8	35.6	3.9
F3*	Things "fall between the cracks" when transferring patients from one unit to another.	3.20	0.80	1.4	16.2	47.2	31.4	3.8
F4	There is good cooperation among hospital units that need to work together.	3.42	0.77	1.0	10.0	39.2	45.7	4.2
F5*	Important patient care information is often lost during shift changes.	3.52	0.74	0.6	6.9	38.2	48.0	6.2
F6*	It is often unpleasant to work with staff from other hospital units.	3.57	0.87	1.4	9.9	30.2	46.9	11.6
F7*	Problems often occur in the exchange of information across hospital units.	3.54	0.77	0.6	8.3	34.6	49.3	7.2
F8	The actions of hospital management show that patient safety is a top priority.	3.46	0.82	1.7	9.1	37.5	44.9	6.8
F9*	Hospital management seems interested in patient safety only after an adverse event happens.	3.76	0.78	0.7	5.2	25.2	54.8	14.0
F10	Hospital units work well together to provide the best care for patients.	3.46	0.79	1.4	9.3	36.6	47.3	5.4
F11*	Shift changes are problematic for patients in this hospital.	3.72	0.71	0.4	2.7	32.1	53.7	11.1

*, Responses to negatively worded items were reversed as follows: 1 to 5, 2 to 4, 4 to 2, and 5 to 1; SD, Standard deviation; n=1,756.

Table S4 Descriptive statistics of responses to the psychological safety survey

Codes	Items	Mean	SD	Proportion of responses (%)						
				1	2	3	4	5	6	7
About the team you usually work with…										
H1*	If you make a mistake on this team, it is often held against you.	3.35	1.39	4.7	26.7	27.6	22.5	9.2	7.0	2.3
H2	Members of this team are able to bring up problems and tough issues.	5.00	1.10	0.7	2.0	6.3	18.1	37.9	30.5	4.4
H3	People on this team sometimes reject others for being different.	4.43	1.19	1.3	4.5	13.1	33.5	28.5	16.8	2.3
H4	It is safe to take a risk on this team.	4.91	1.17	1.2	2.2	7.1	21.8	34.7	27.7	5.4
H5*	It is difficult to ask other members of this team for help.	5.03	1.22	0.9	2.7	7.3	15.9	37.1	26.6	9.5
H6	No one on this team would deliberately act in a way that undermines my efforts.	5.24	1.37	1.0	4.0	6.2	16.9	17.5	39.6	14.7
H7	Working with members of this team, my unique skills and talents are valued and utilized.	4.81	1.20	1.5	2.9	5.4	28.3	32.8	23.1	5.9

*, Responses to negatively worded items were reversed as follows: 1 to 7, 2 to 6, 3 to 5, 5 to 3, 6 to 2, and 7 to 1; SD, Standard deviation; n=1,756.

Table S5 Descriptive statistics of responses to the work engagement survey

Codes	Items	Mean	SD	Proportion of responses (%)						
				1	2	3	4	5	6	7
About your work (currently in charge) …										
I1	At my work, I feel bursting with energy.	3.99	1.80	12.9	10.4	12.4	26.5	11.3	19.2	7.2
I2	At my job I feel strong and vigorous.	3.97	1.82	12.8	11.3	12.8	26.0	10.8	18.2	8.0
I3	I am enthusiastic about my job.	5.38	1.66	3.6	4.7	4.6	16.7	9.9	28.9	31.7
I4	My job inspires me.	4.27	1.87	12.0	9.2	10.1	22.7	12.5	22.0	11.5
I5	When I get up in the morning, I feel like going to work.	3.96	2.10	21.3	8.3	10.8	17.4	10.3	18.4	13.6
I6	I feel happy when I am working intensely.	3.63	1.96	22.3	10.8	13.4	19.4	10.8	16.2	7.2
I7	I am proud on the work that I do.	4.86	1.79	5.0	7.6	9.1	21.6	10.7	23.1	22.8
I8	I am immersed in my work.	4.01	1.94	17.1	8.0	12.5	21.1	11.0	20.7	9.5
I9	I get carried away when I am working.	4.25	1.97	14.4	8.1	11.2	19.6	10.9	22.2	13.6

*, Responses to negatively worded items were reversed as follows: 1 to 7, 2 to 6, 3 to 5, 5 to 3, 6 to 2, and 7 to 1; SD, Standard deviation; n=1,756.

Table S6 Confirmatory factor analysis in patient safety culture

Factors	Codes	Items	Factor loadings
1. Teamwork Within Units	A1	People support one another in this unit.	0.820
	A3	When a lot of work needs to be done quickly, we work together as a team to get the work done.	0.812
	A4	In this unit, people treat each other with respect.	0.793
	A11	When one area in this unit gets really busy, others help out.	0.452
2. Supervisor/Manager Expectations & Actions Promoting Patient Safety	B1	My supervisor/manager says a good word when he/she sees a job done according to established patient safety procedures.	0.662
	B2	My supervisor/manager seriously considers staff suggestions for improving patient safety.	0.798
	B3*	Whenever pressure builds up, my supervisor/manager wants us to work faster, even if it means taking shortcuts.	0.569
	B4*	My supervisor/manager overlooks patient safety problems that happen over and over.	0.671
3. Organizational Learning–Continuous Improvement	A6	We are actively doing things to improve patient safety.	0.655
	A9	Mistakes have led to positive changes here.	0.607
	A13	After we make changes to improve patient safety, we evaluate their effectiveness.	0.565
4. Management Support for Patient Safety	F1	Hospital management provides a work climate that promotes patient safety.	0.667
	F8	The actions of hospital management show that patient safety is a top priority.	0.567
	F9*	Hospital management seems interested in patient safety only after an adverse event happens.	0.687
5. Overall Perceptions of Patient Safety	A15	Patient safety is never sacrificed to get more work done.	0.519
	A18	Our procedures and systems are good at preventing errors from happening.	0.605
	A10*	It is just by chance that more serious mistakes don't happen around here.	0.606
	A17*	We have patient safety problems in this unit.	0.712
6. Feedback & Communication About Error	C1	We are given feedback about changes put into place based on event reports.	0.712
	C3	We are informed about errors that happen in this unit.	0.757
	C5	In this unit, we discuss ways to prevent errors from happening again.	0.803
7. Communication Openness	C2	Staff will freely speak up if they see something that may negatively affect patient care.	0.760
	C4	Staff feel free to question the decisions or actions of those with more authority.	0.756
	C6*	Staff are afraid to ask questions when something does not seem right.	0.674
8. Frequency of Events Reported	D1	When a mistake is made, but is caught and corrected before affecting the patient, how often is this reported?	0.766
	D2	When a mistake is made, but has no potential to harm the patient, how often is this reported?	0.956
	D3	When a mistake is made that could harm the patient, but does not, how often is this reported?	0.801
9. Teamwork Across Units	F4	There is good cooperation among hospital units that need to work together.	0.687
	F10	Hospital units work well together to provide the best care for patients.	0.693
	F2*	Hospital units do not coordinate well with each other.	0.674
	F6*	It is often unpleasant to work with staff from other hospital units.	0.620
10. Staffing	A2	We have enough staff to handle the workload.	0.531
	A5*	Staff in this unit work longer hours than is best for patient care.	0.526
	A7*	We use more agency/temporary staff than is best for patient care.	0.466
	A14*	We work in "crisis mode" trying to do too much, too quickly.	0.675
11. Handoffs & Transitions	F3*	Things "fall between the cracks" when transferring patients from one unit to another.	0.664
	F5*	Important patient care information is often lost during shift changes.	0.692
	F7*	Problems often occur in the exchange of information across hospital units.	0.710
	F11*	Shift changes are problematic for patients in this hospital.	0.610
12. Nonpunitive Response to Error	A8*	Staff feel like their mistakes are held against them.	0.774
	A12*	When an event is reported, it feels like the person is being written up, not the problem.	0.706
	A16*	Staff worry that mistakes they make are kept in their personnel file.	0.594

*, Responses to negatively worded items were reversed as follows: 1 to 5, 2 to 4, 4 to 2, and 5 to 1; Comparative fit index (CFI), 0.911; Tucker-Lewis index (TLI), 0.899; Root mean square error of approximation (RMSEA), 0.046; Standardized root mean square residual (SRMR), 0.041; n=1,756.

Table S7 Internal reliability of the 12 subscales of the patient safety culture scale

Factors	Cronbach's alpha	McDonald's omega
1. Teamwork Within Units	0.78	0.82
2. Supervisor/Manager Expectations & Actions Promoting Patient Safety	0.76	0.77
3. Organizational Learning—Continuous Improvement	0.63	0.64
4. Management Support for Patient Safety	0.67	0.68
5. Overall Perceptions of Patient Safety	0.69	0.71
6. Feedback & Communication About Error	0.80	0.80
7. Communication Openness	0.77	0.78
8. Frequency of Events Reported	0.87	0.88
9. Teamwork Across Units	0.76	0.76
10. Staffing	0.64	0.64
11. Handoffs & Transitions	0.76	0.76
12. Nonpunitive Response to Errors	0.73	0.73

*, Responses to negatively worded items were reversed as follows: 1 to 5, 2 to 4, 4 to 2, and 5 to 1; n=1,756.

Table S8 Confirmatory factor analysis in psychological safety

Factor	Codes	Items	Factor loadings
1. Psychological Safety	H1*	If you make a mistake on this team, it is often held against you.	0.247
	H2	Members of this team are able to bring up problems and tough issues.	-0.684
	H3	People on this team sometimes reject others for being different.	-0.605
	H4	It is safe to take a risk on this team.	-0.844
	H5*	It is difficult to ask other members of this team for help.	-0.608
	H6	No one on this team would deliberately act in a way that undermines my efforts.	-0.596
	H7	Working with members of this team, my unique skills and talents are valued and utilized.	-0.791

*, Responses to negatively worded items were reversed as follows: 1 to 7, 2 to 6, 3 to 5, 5 to 3, 6 to 2, and 7 to 1; Comparative fit index (CFI), 0.926; Tucker-Lewis index (TLI), 0.889; Root mean square error of approximation (RMSEA), 0.112; Standardized root mean square residual (SRMR), 0.054; n=1,756.

Table S9 Exploratory factor analysis in psychological safety

Codes	Items	Factor 1	Communality
H4	It is safe to take a risk on this team.	0.842	0.709
H7	Working with members of this team, my unique skills and talents are valued and utilized.	0.796	0.634
H2	Members of this team are able to bring up problems and tough issues.	0.675	0.455
H5*	It is difficult to ask other members of this team for help.	0.613	0.376
H3	People on this team sometimes reject others for being different.	0.602	0.362
H6	No one on this team would deliberately act in a way that undermines my efforts.	0.601	0.361
	Eigenvalues	3.448	
	Factor contribution	2.896	
	Variance explained	0.483	
	Cronbach's alpha	0.84	
	McDonald's omega	0.85	

*, Responses to negatively worded items were reversed as follows: 1 to 7, 2 to 6, 3 to 5, 5 to 3, 6 to 2, and 7 to 1; n=1,756.

Table S10 Confirmatory factor analysis in work engagement

Factors	Codes	Items	Factor loadings
1. Vigor	I1	At my work, I feel bursting with energy.	0.956
	I2	At my job I feel strong and vigorous.	0.961
	I5	When I get up in the morning, I feel like going to work.	0.749
2. Dedication	I3	I am enthusiastic about my job.	0.624
	I4	My job inspires me.	0.913
	I7	I am proud on the work that I do.	0.745
3. Absorption	I6	I feel happy when I am working intensely.	0.852
	I8	I am immersed in my work.	0.858
	I9	I get carried away when I am working.	0.828

Comparative fit index (CFI), 0.909; Tucker-Lewis index (TLI), 0.863; Root mean square error of approximation (RMSEA), 0.180; Standardized root mean square residual (SRMR), 0.062; n=1,756.

Table S11 Exploratory factor analysis in work engagement

Codes	Items	Factor 1	Communality
I2	At my job I feel strong and vigorous.	0.925	0.856
I1	At my work, I feel bursting with energy.	0.924	0.854
I4	My job inspires me.	0.906	0.820
I6	I feel happy when I am working intensely.	0.861	0.741
I5	When I get up in the morning, I feel like going to work.	0.790	0.625
I8	I am immersed in my work.	0.759	0.576
I9	I get carried away when I am working.	0.716	0.512
I7	I am proud on the work that I do.	0.715	0.511
	Eigenvalues	6.280	
	Factor contribution	5.496	
	Variance explained	0.687	
	Cronbach's alpha	0.95	
	McDonald's omega	0.95	

n=1,756

Table S12 Pearson correlation matrix among the 14 factor scores

Factors	f1	f2	f3	f4	f5	f6	f7	f8	f9	f10	f11	f12	f13	f14
f1	1	0.543	0.565	0.464	0.503	0.480	0.571	0.291	0.498	0.271	0.396	0.463	0.628	0.357
f2	0.543	1	0.508	0.479	0.549	0.536	0.596	0.332	0.413	0.310	0.431	0.516	0.583	0.356
f3	0.565	0.508	1	0.506	0.514	0.574	0.511	0.305	0.435	0.180	0.417	0.332	0.522	0.312
f4	0.464	0.479	0.506	1	0.571	0.476	0.476	0.280	0.652	0.305	0.602	0.361	0.459	0.340
f5	0.503	0.549	0.514	0.571	1	0.442	0.515	0.291	0.495	0.481	0.540	0.541	0.525	0.350
f6	0.480	0.536	0.574	0.476	0.442	1	0.622	0.440	0.365	0.204	0.411	0.343	0.506	0.239
f7	0.571	0.596	0.511	0.476	0.515	0.622	1	0.324	0.428	0.265	0.429	0.542	0.659	0.364
f8	0.291	0.332	0.305	0.280	0.291	0.440	0.324	1	0.244	0.123	0.294	0.227	0.316	0.102
f9	0.498	0.413	0.435	0.652	0.495	0.365	0.428	0.244	1	0.246	0.638	0.360	0.445	0.324
f10	0.271	0.310	0.180	0.305	0.481	0.204	0.265	0.123	0.246	1	0.332	0.448	0.288	0.271
f11	0.396	0.431	0.417	0.602	0.540	0.411	0.429	0.294	0.638	0.332	1	0.396	0.439	0.273
f12	0.463	0.516	0.332	0.361	0.541	0.343	0.542	0.227	0.360	0.448	0.396	1	0.530	0.327
f13	0.628	0.583	0.522	0.459	0.525	0.506	0.659	0.316	0.445	0.288	0.439	0.530	1	0.449
f14	0.357	0.356	0.312	0.340	0.350	0.239	0.364	0.102	0.324	0.271	0.273	0.327	0.449	1

f1–f12, 12 subscales of the Hospital Survey on Patient Safety Culture; f13, Psychological Safety; f14, Work Engagement; n=1,756.

Table S13 Analysis of inter-factor relationships by multilevel structural equation modeling (individual level)

Affecting variables		Affected variables	Estimates	SE	95%CI	Standardized estimates	*p*-value
13. Psychological Safety	→	1. Teamwork Within Units	0.41	0.01	0.38–0.44	0.61	<0.001*
		2. Supervisor/Manager Expectations & Actions Promoting Patient Safety	0.37	0.01	0.34–0.39	0.56	<0.001*
		3. Organizational Learning—Continuous Improvement	0.28	0.01	0.25–0.30	0.50	<0.001*
		4. Management Support for Patient Safety	0.33	0.02	0.30–0.36	0.47	<0.001*
		5. Overall Perceptions of Patient Safety	0.33	0.02	0.30–0.36	0.50	<0.001*
		6. Feedback & Communication About Error	0.39	0.02	0.35–0.42	0.52	<0.001*
		7. Communication Openness	0.51	0.02	0.47–0.54	0.63	<0.001*
		8. Frequency of Events Reported	0.27	0.02	0.22–0.31	0.29	<0.001*
		9. Teamwork Across Units	0.36	0.02	0.32–0.39	0.48	<0.001*
		10. Staffing	0.23	0.02	0.19–0.27	0.31	<0.001*
		11. Handoffs & Transitions	0.28	0.02	0.25–0.31	0.43	<0.001*
		12. Nonpunitive Response to Errors	0.41	0.02	0.37–0.45	0.48	<0.001*
13. Psychological Safety	→	14. Work Engagement	0.78	0.04	0.70–0.87	0.43	<0.001*

SE, standard error; CI, confidence interval; Comparative fit index (CFI), 0.964; Tucker-Lewis index (TLI), 0.910; Root mean square error of approximation (RMSEA), 0.057; Standardized root mean square residual (SRMR), 0.000 (within); *, $p<0.05$; n=1,446.

Table S14 Analysis of inter-factor and incident count relationships by multilevel structural equation modeling (department level)

Affecting variables		Affected variables	Estimates	SE	95%CI	Standardized estimates	*p*-value
13. Psychological Safety	→	1. Teamwork Within Units	0.46	0.05	0.36–0.55	0.70	<0.001*
		2. Supervisor/Manager Expectations & Actions Promoting Patient Safety	0.55	0.04	0.47–0.63	0.81	<0.001*
		3. Organizational Learning—Continuous Improvement	0.40	0.04	0.31–0.48	0.68	<0.001*
		4. Management Support for Patient Safety	0.29	0.05	0.18–0.39	0.48	<0.001*
		5. Overall Perceptions of Patient Safety	0.46	0.05	0.36–0.55	0.70	<0.001*
		6. Feedback & Communication About Error	0.43	0.07	0.29–0.56	0.55	<0.001*
		7. Communication Openness	0.57	0.04	0.49–0.65	0.82	<0.001*
		8. Frequency of Events Reported	0.33	0.08	0.16–0.49	0.37	<0.001*
		9. Teamwork Across Units	0.23	0.06	0.11–0.35	0.36	<0.001*
		10. Staffing	0.18	0.09	0.00–0.37	0.20	0.048*
		11. Handoffs & Transitions	0.36	0.06	0.24–0.48	0.53	<0.001*
		12. Nonpunitive Response to Errors	0.58	0.05	0.49–0.67	0.78	<0.001*
13. Psychological Safety	→	14. Work Engagement	0.98	0.14	0.71–1.26	0.59	<0.001*
1. Teamwork Within Units	→	Number of incidents	1.00	0.89	-0.74–2.75	0.15	0.260
2. Supervisor/Manager Expectations & Actions Promoting Patient Safety			2.95	0.90	1.19–4.71	0.47	0.001*
3. Organizational Learning—Continuous Improvement			0.97	1.05	-1.09–3.02	0.13	0.357
4. Management Support for Patient Safety			-1.99	1.22	-4.37–0.40	-0.28	0.102
5. Overall Perceptions of Patient Safety			0.65	1.06	-1.42–2.73	0.10	0.537
6. Feedback & Communication About Error			2.22	0.75	0.74–3.69	0.41	0.003*
7. Communication Openness			-0.90	1.02	-2.91–1.11	-0.15	0.380
8. Frequency of Events Reported			-0.08	0.50	-1.05–0.89	-0.02	0.878
9. Teamwork Across Units			1.05	0.88	-0.66–2.77	0.16	0.229
10. Staffing			-0.46	0.46	-1.36–0.43	-0.10	0.308
11. Handoffs & Transitions			2.30	0.81	0.71–3.88	0.37	0.005*
12. Nonpunitive Response to Errors			-1.64	0.86	-3.33–0.04	-0.29	0.056
13. Psychological Safety			-1.96	0.93	-3.78–-0.14	-0.46	0.035*
14. Work Engagement			-0.36	0.27	-0.88–0.16	-0.14	0.170

SE, standard error; CI, confidence interval; Comparative fit index (CFI), 0.964; Tucker-Lewis index (TLI), 0.910; Root mean square error of approximation (RMSEA), 0.057; Standardized root mean square residual (SRMR), 0.163 (between); *, $p<0.05$; n=94.

Table S15 Analysis of inter-factor relationships by multilevel structural equation modeling (individual level)

Affecting variables		Affected variables	Estimates	SE	95%CI	Standardized estimates	*p*-value
13. Psychological Safety	→	1. Teamwork Within Units	0.41	0.01	0.38–0.44	0.61	<0.001*
		2. Supervisor/Manager Expectations & Actions Promoting Patient Safety	0.37	0.01	0.34–0.39	0.56	<0.001*
		3. Organizational Learning—Continuous Improvement	0.28	0.01	0.26–0.30	0.50	<0.001*
		4. Management Support for Patient Safety	0.33	0.02	0.30–0.36	0.47	<0.001*
		5. Overall Perceptions of Patient Safety	0.33	0.02	0.30–0.36	0.50	<0.001*
		6. Feedback & Communication About Error	0.39	0.02	0.36–0.42	0.52	<0.001*
		7. Communication Openness	0.51	0.02	0.48–0.54	0.64	<0.001*
		8. Frequency of Events Reported	0.26	0.02	0.22–0.31	0.29	<0.001*
		9. Teamwork Across Units	0.36	0.02	0.33–0.39	0.48	<0.001*
		10. Staffing	0.23	0.02	0.20–0.27	0.31	<0.001*
		11. Handoffs & Transitions	0.28	0.02	0.25–0.31	0.43	<0.001*
		12. Nonpunitive Response to Errors	0.41	0.02	0.37–0.45	0.48	<0.001*
13. Psychological Safety	→	14. Work Engagement	0.79	0.04	0.70–0.87	0.42	<0.001*

SE, standard error; CI, confidence interval; Comparative fit index (CFI), 0.964; Tucker-Lewis index (TLI), 0.908; Root mean square error of approximation (RMSEA), 0.058; Standardized root mean square residual (SRMR), 0.000 (within); *, $p<0.05$; n=1,478.

Table S16 Analysis of inter-factor and incident count relationships by multilevel structural equation modeling (department level)

Affecting variables		Affected variables	Estimates	SE	95%CI	Standardized estimates	*p*-value
13. Psychological Safety	→	1. Teamwork Within Units	0.44	0.05	0.35–0.53	0.70	<0.001*
		2. Supervisor/Manager Expectations & Actions Promoting Patient Safety	0.53	0.04	0.45–0.61	0.80	<0.001*
		3. Organizational Learning—Continuous Improvement	0.40	0.04	0.31–0.48	0.70	<0.001*
		4. Management Support for Patient Safety	0.28	0.05	0.19–0.37	0.52	<0.001*
		5. Overall Perceptions of Patient Safety	0.46	0.05	0.37–0.55	0.72	<0.001*
		6. Feedback & Communication About Error	0.41	0.06	0.29–0.54	0.56	<0.001*
		7. Communication Openness	0.56	0.04	0.49–0.63	0.85	<0.001*
		8. Frequency of Events Reported	0.33	0.08	0.17–0.48	0.39	<0.001*
		9. Teamwork Across Units	0.23	0.06	0.11–0.35	0.36	<0.001*
		10. Staffing	0.20	0.09	0.03–0.37	0.23	0.022*
		11. Handoffs & Transitions	0.34	0.06	0.22–0.45	0.52	<0.001*
		12. Nonpunitive Response to Errors	0.56	0.05	0.47–0.65	0.79	<0.001*
13. Psychological Safety	→	14. Work Engagement	0.98	0.13	0.72–1.23	0.61	<0.001*
1. Teamwork Within Units	→	Number of incidents	1.98	0.54	0.92–3.04	0.51	<0.001*
2. Supervisor/Manager Expectations & Actions Promoting Patient Safety			1.86	0.52	0.85–2.88	0.51	<0.001*
3. Organizational Learning—Continuous Improvement			-0.43	0.67	-1.75–0.88	-0.10	0.518
4. Management Support for Patient Safety			0.01	0.78	-1.52–1.53	0.00	0.993
5. Overall Perceptions of Patient Safety			0.23	0.61	-0.96–1.43	0.06	0.701
6. Feedback & Communication About Error			0.64	0.44	-0.21–1.50	0.19	0.140
7. Communication Openness			-0.56	0.68	-1.89–0.78	-0.15	0.415
8. Frequency of Events Reported			0.15	0.30	-0.44–0.75	0.05	0.610
9. Teamwork Across Units			-0.95	0.51	-1.95–0.04	-0.25	0.060
10. Staffing			-0.71	0.27	-1.24–-0.19	-0.25	0.008*
11. Handoffs & Transitions			1.54	0.50	0.55–2.52	0.42	0.002*
12. Nonpunitive Response to Errors			0.10	0.56	-1.00–1.20	0.03	0.863
13. Psychological Safety			-2.27	0.55	-3.35–-1.19	-0.94	<0.001*
14. Work Engagement			0.23	0.16	-0.09–0.55	0.15	0.158

SE, standard error; CI, confidence interval; Comparative fit index (CFI), 0.964; Tucker-Lewis index (TLI), 0.908; Root mean square error of approximation (RMSEA), 0.058; Standardized root mean square residual (SRMR), 0.158 (between); *, $p<0.05$; n=91.

Table S17 Analysis of inter-factor and incident count relationships by multiple regression analysis (department level)

Affecting variables		Affected variables	Estimates	SE	95%CI	Standardized estimates	*p*-value	VIF
1. Teamwork Within Units	→	Number of incidents	3.09	1.26	0.59–5.59	0.34	0.016*	2.99
2. Supervisor/Manager Expectations & Actions Promoting Patient Safety			5.25	1.28	2.70–7.81	0.60	<0.001*	3.41
3. Organizational Learning—Continuous Improvement			-0.12	1.55	-3.21–2.96	-0.01	0.936	3.68
4. Management Support for Patient Safety			-1.27	1.76	-4.77–2.24	-0.13	0.475	4.87
5. Overall Perceptions of Patient Safety			0.00	1.50	-2.99–2.99	0.00	0.999	4.47
6. Feedback & Communication About Error			2.46	1.12	0.24–4.68	0.32	0.030*	3.42
7. Communication Openness			-1.72	1.51	-4.72–1.28	-0.20	0.256	4.90
8. Frequency of Events Reported			-0.31	0.73	-1.77–1.15	-0.05	0.673	1.89
9. Teamwork Across Units			-0.67	1.23	-3.11–1.78	-0.07	0.588	2.93
10. Staffing			-1.30	0.66	-2.60–0.01	-0.20	0.052	1.64
11. Handoffs & Transitions			4.08	1.16	1.77–6.39	0.47	0.001*	2.85
12. Nonpunitive Response to Errors			-1.28	1.27	-3.81–1.26	-0.16	0.319	3.94
13. Psychological Safety			-3.99	1.27	-6.51–-1.47	-0.68	0.002*	7.41
14. Work Engagement			-0.04	0.38	-0.81–0.72	-0.01	0.910	1.92

SE, standard error; CI, confidence interval; VIF, variance inflation factor; R^2, 0.479; Adjusted R^2, 0.392; $F(14, 83) = 5.46$, $p<0.001$; *, $p<0.05$; n=98.

Supplemental Figures

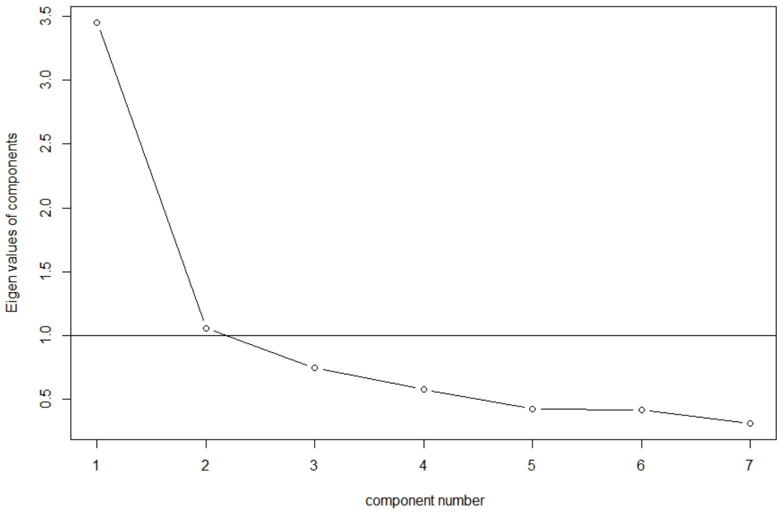

Figure S1 Scree plot of exploratory factor analysis for psychological safety n=1,756

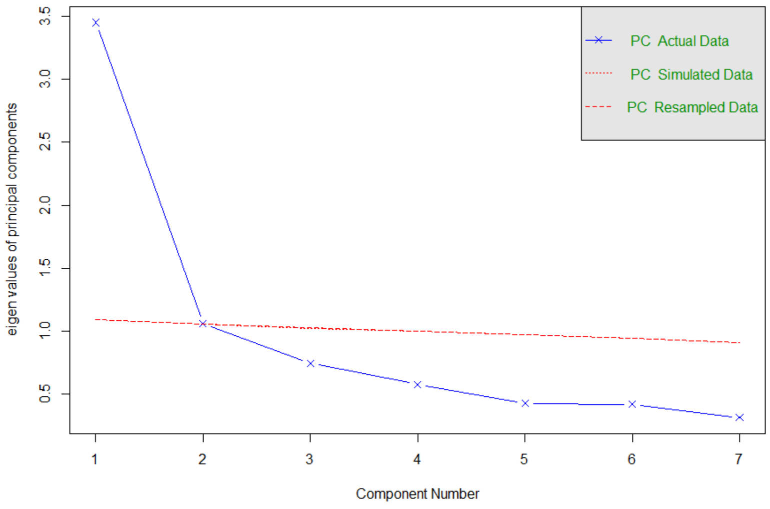

Figure S2 Parallel analysis of exploratory factor analysis for psychological safety
PC, principal components; n=1,756

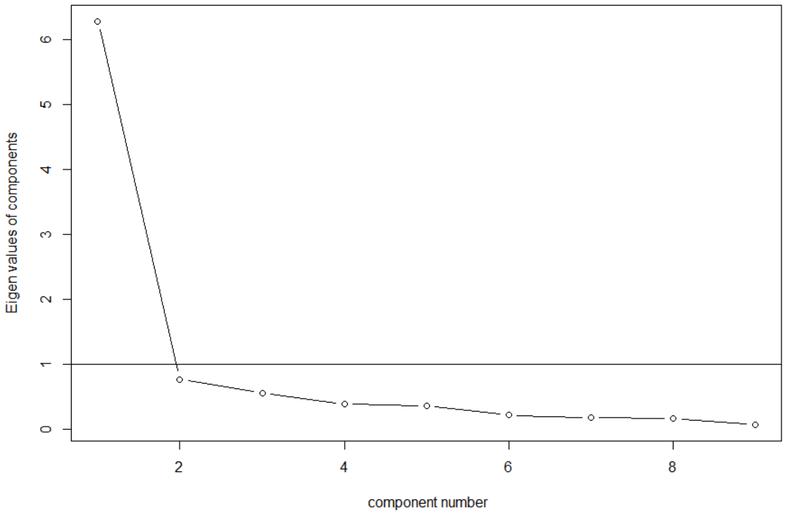

Figure S3 Scree plot of exploratory factor analysis for work engagement n=1,756

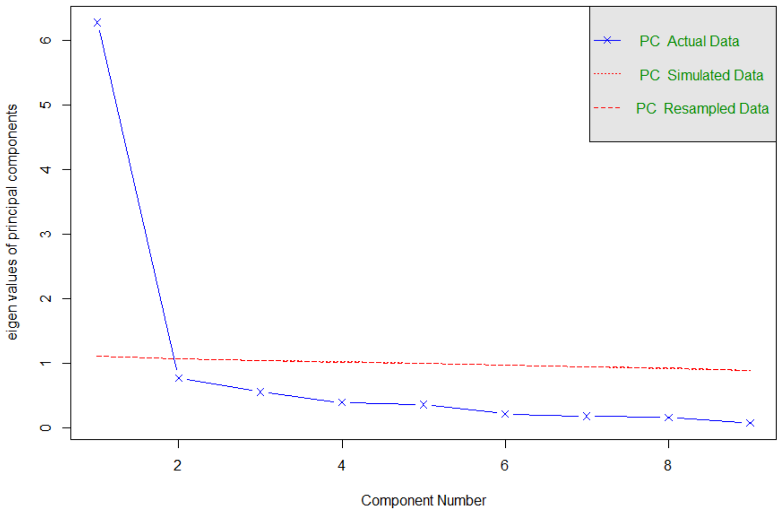

Figure S4 Parallel analysis of exploratory factor analysis for work engagement
PC, principal components; n=1,756

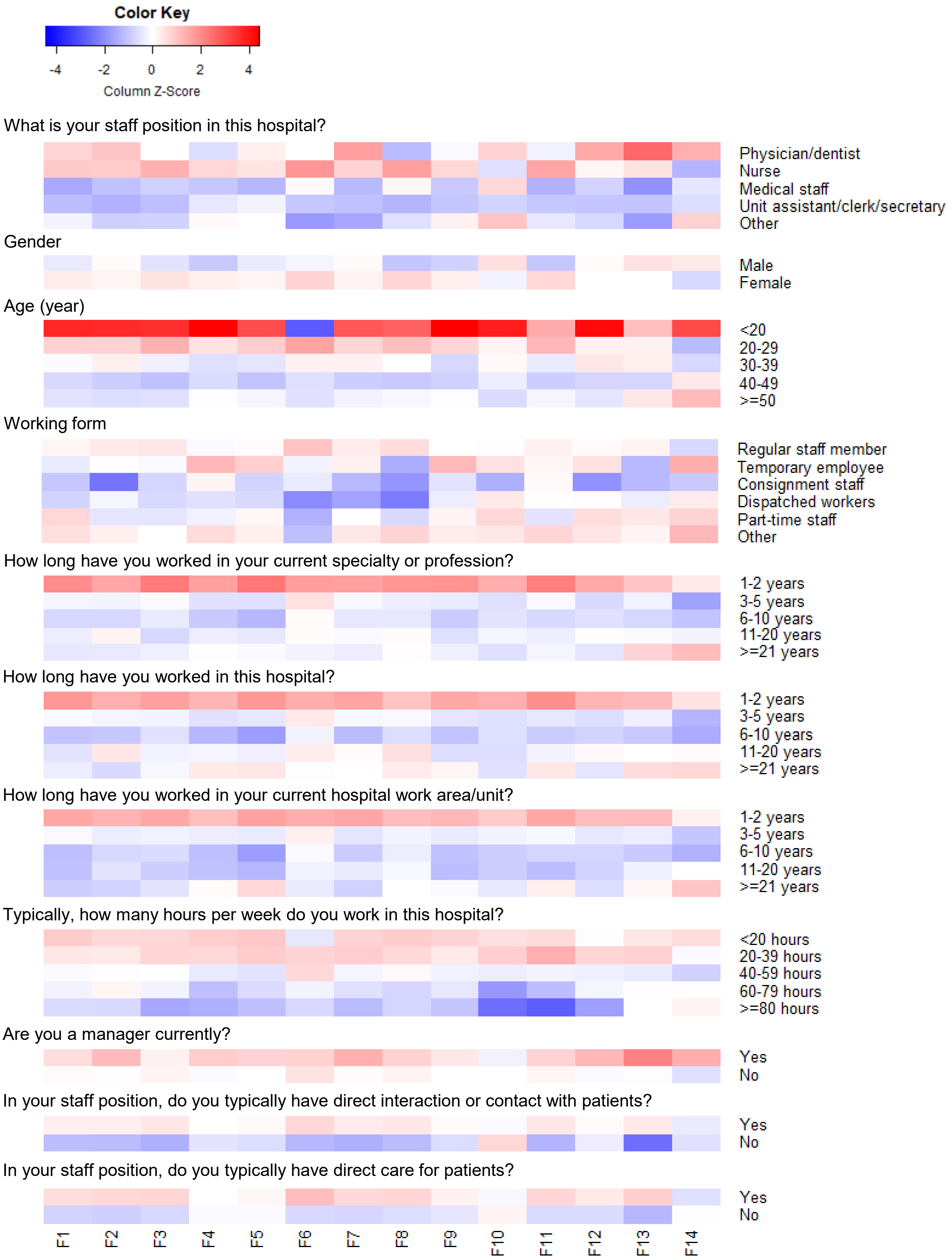

Figure S5 Comparison of mean factor scores by respondent background n=1,756

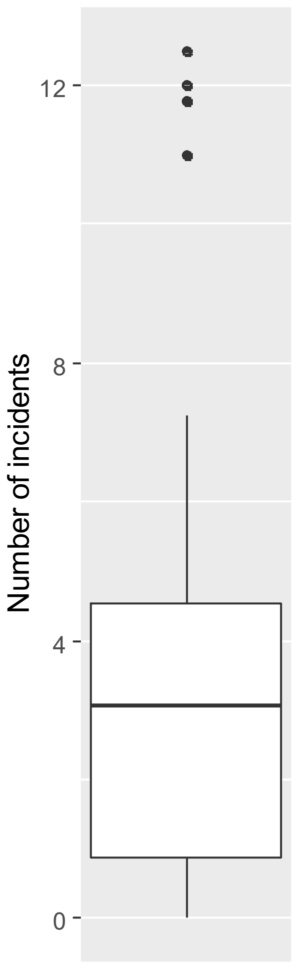

Figure S6 Boxplot of departmental incident counts
The horizontal line in the box represents the median, the box represents the interquartile range, and the whiskers represent the length truncated to the nearest value within 1.5 times the interquartile range. The plots represent outliers. The incident count is adjusted by dividing by the number of personnel in the department.
n=98
